# Is the Hong Kong Liver Cancer staging system the best guide for hepatitis B virus-related hepatocellular carcinoma patients with multiple tumors?

**DOI:** 10.18632/oncotarget.9956

**Published:** 2016-06-13

**Authors:** Shuang Liu, Xiaoqiang Li, Hui Li, Lei Guo, Bo Zhang, Jubo Zhang, Qinghai Ye

**Affiliations:** ^1^ Liver Cancer Institute and Zhongshan Hospital, Fudan University, Key Laboratory of Carcinogenesis and Cancer Invasion, Fudan University, Ministry of Education, Shanghai, China

**Keywords:** hepatocellular carcinoma, multiple tumors, Hong Kong Liver Cancer staging system, radical resection, overall survival

## Abstract

It still must be confirmed whether the newly developed Hong Kong Liver Cancer Staging (HKLC) system can effectively stratify patients with multiple tumors and identify patients who could obtain a survival benefit with radical resection. In this study, we retrospectively compared survival rates of surgery versus transcatheter arterial chemoembolization for hepatitis B virus-related hepatocellular carcinoma patients with multiple tumors by using the propensity score method. In addition, the prognostic roles of tumor size, number and thrombus status together with other covariates on postoperative survival were analyzed by multivariate analysis. In matched cohorts, surgical treatment could significantly reduce patient mortality in patients within or outside HKLC criteria (odds ratio (OR) = 0.5, *P* < 0.001, OR = 0.6, *P* = 0.001, respectively). In 941 patients undergoing radical resection, the state of tumor thrombus demonstrated a significant interaction with tumor size on postoperative survival (*P* for interaction = 0.041). Tumor number was not a predictor of postoperative survival in patients with multiple tumors (adjusted OR = 1.1, *P* = 0.202). In patients without tumor thrombus, tumor size > 5 cm was an independent risk factor of postoperative survival (OR = 1.7, *P* < 0.001). In patients without tumor thrombus, patient survival was mainly influenced by tumor location (OR = 2.1, *P* < 0.001). In summary, patients with multiple tumors could obtain a survival benefit from radical surgery based on the more aggressive HKLC staging system. However, parameters in this staging system still need further adjustments.

## INTRODUCTION

In the treatment of hepatocellular carcinoma (HCC), the Barcelona Clinic Liver Cancer (BCLC) staging classification has been widely adopted because it links prognostic classification to treatment recommendations [1]. Because the BCLC staging classification was developed in patients from western countries with predominantly alcoholic liver disease and hepatitis C-related HCC, it might not be able to provide accurate outcome predictions and appropriate treatment options in HCC patients for whom hepatitis B virus (HBV) infection is the predominant etiologic factor. Recently, a new staging system called the Hong Kong Liver Cancer (HKLC) criteria was developed, which might be more applicable to HBV-related HCC patients [2].

For surgeons, the vast difference between the HKLC criteria and conventional BCLC criteria lies in the treatment of patients with multiple tumors. For these patients, the BCLC system recommends transcatheter arterial chemoembolization (TACE), liver transplantation or sorafenib depending on the state of tumor thrombus. However, the HKLC system attempts to stratify patients with multiple tumors by the size of the largest tumor in the liver (≤ 5 or > 5 cm), number of tumor nodules (≤ 3 or > 3) and tumor thrombus (yes or no) status to identify patients who could obtain survival benefits from radical resection and recommend surgeons to carry out the procedure [2]. With advancements in technology and surgical methods, the risks of the operation have decreased gradually and many surgeons also strive for the opportunity to cure these patients. A significant proportion of patients with multiple tumors also would choose surgical resection for a potential cure. However, whether these more aggressive criteria could bring a survival benefit to HBV-related HCC patients still remains to be confirmed.

In the present study, we retrospectively analyzed the survival of HBV-related HCC patients with multiple tumors undergoing surgery and TACE. Then, we compared patient overall survival according to the HKLC criteria to validate the guiding role of this system for surgeons. We also checked the parameters enrolled in the HKLC system to make the criteria proposed more scientific. Tough this study, we expect to provide the best treatment options for patients with multiple HBV-related HCC so that patients can experience longer survival.

## RESULTS

Baseline characteristics of the two propensity score-matched groups stratified by HKLC criteria were showed in Table 1. Surgery and TACE groups were well balanced for all covariates, including age, sex, total bilirubin, alanine aminotransferase (ALT), gamma-glutamyl transferase (GGT), alpha-fetoprotein (AFP), tumor size, number, thrombus and location, either in patients within or outside HKLC criteria (all, P > 0.05).

**Table 1:** Baseline characteristics of the two propensity-matched groups stratified by HKLC criteria

	Within HKLC criteria			Outside HKLC criteria		
	Surgery	TACE	*P*	Surgery	TACE	*P*
	(n = 237)	(n = 237)		(n = 117)	(n = 117)	
Age, years			0.403			0.226
≤ 60	171 (72.2)	179 (75.5)		84 (71.8)	92 (78.6)	
> 60	66 (27.8)	58 (24.5)		33 (28.2)	25 (21.4)	
Sex			0.882			0.306
Female	26 (11.0)	25 (10.5)		11 (9.4)	16 (13.7)	
Male	211 (89.0)	212 (89.5)		106 (90.6)	101 (86.3)	
Total bilirubin, μmol/L			0.831			0.851
≤ 20.4	178 (75.1)	180 (75.9)		100 (85.5)	101 (86.3)	
> 20.4	59 (24.9)	57 (24.1)		17 (14.5)	16 (13.7)	
Serum ALT, u/L			0.456			0.353
≤ 42	175 (73.8)	182 (76.8)		72 (61.5)	65 (55.6)	
> 42	62 (26.2)	55 (23.2)		45 (38.5)	52 (44.4)	
Serum GGT, u/L			0.565			0.420
≤ 54	87 (36.7)	81 (34.2)		48 (41.0)	42 (35.9)	
> 54	150 (63.3)	156 (65.8)		69 (59.0)	75 (64.1)	
Serum AFP, ng/mL			0.412			0.307
≤ 20	62 (26.2)	70 (29.5)		24 (20.5)	18 (15.4)	
> 20	175 (73.8)	167 (70.5)		93 (79.5)	99 (84.6)	
Tumor size, cm			1.000			1.000
≤ 5	149 (62.9)	149 (62.9)		9 (7.7)	9 (7.7)	
> 5	88 (37.1)	88 (37.1)		108 (92.3)	108 (92.3)	
Tumor number			1.000			0.592
≤ 3	212 (89.5)	212 (89.5)		48 (41.00%)	44 (37.60%)	
> 3	25 (10.5)	25 (10.5)		69 (59.00%)	73 (62.40%)	
Tumor thrombus			1.000			0.346
Without	217 (91.6)	217 (91.6)		41 (35.00%)	48 (41.00%)	
With	20 (8.4)	20 (8.4)		76 (65.00%)	69 (59.00%)	
Tumor location			0.266			0.540
One lobe	140 (59.1)	128 (54.0)		26 (22.2)	30 (25.6)	
More than one lobe	97 (40.9)	109 (46.0)		91 (77.8	87 (74.4)	

Abbreviations: TACE, transcatheter arterial chemoembolization; ALT: alanine aminotransferase; GGT, gamma-glutamyl transferase; AFP, alpha-fetoprotein.

All of the covariates showed in Table 1 were incorporated into multivariate Cox regression analyses and independent risk factors associated with patient survival in each subgroup are reported in Table 2. Surgical treatment could significantly reduce patient's mortality for patients within or outside the HKLC criteria (OR = 0.5, *P* < 0.001, OR = 0.6, *P* < 0.001, respectively). In patients within the HKLC criteria, male, tumor size > 5 cm and with tumor thrombus were also independent risk factors of patient survival (OR = 1.8, P = 0.013, OR = 2.0, *P* < 0.001, OR = 1.7, P = 0.031, respectively). In contrast, AFP > 20 ng/ml and tumor location in more than one lobe were significant factor in patients outside the HKLC criteria (OR = 1.6, P = 0.048, OR = 1.6, *P* = 0.013, respectively).

**Table 2:** Multivariate Cox regression analyses of the overall survival stratified by HKLC criteria

	OR(%95 CI)	*P*
**Within HKLC criteria**		
Therapy (surgery vs. TACE)	0.5 (0.4, 0.6)	<0.001*
Sex (male vs. female)	1.8 (1.1, 2.8)	0.013*
Tumor size, cm (> 5 vs. ≤ 5)	2.0 (1.5, 2.6)	<0.001*
Tumor thrombus (with vs. without)	1.7 (1.0, 2.7)	0.031*
**Outside HKLC criteria**		
Therapy (surgery vs. TACE)	0.6 (0.4, 0.8)	0.001*
AFP, ng/mL (> 20 vs. ≤ 20)	1.6 (1.0, 2.5)	0.048*
Tumor location (more than one vs. one lobe)	1.6 (1.1, 2.4)	0.013*

* Significant difference.

Abbreviations: OR: odds ratio; CI: confidence interval; ALT: alanine aminotransferase; GGT, gamma-glutamyl transferase; AFP, Alpha-fetoprotein; TACE, transcatheter arterial chemoembolization;

**Figure 1:**
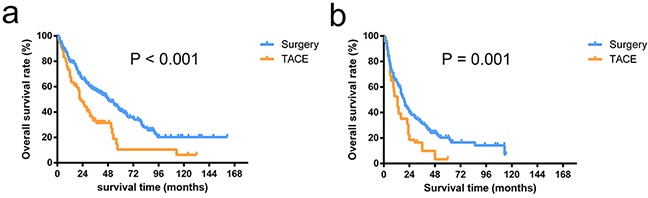
Overall survival curves of patients stratified by HKLC criteria The overall survival rates in patients undergoing surgery were significantly higher than the patients receiving TACE in patients within HKLC criteria (*P* < 0.001) **a.** and outside HKLC criteria (*P* = 0.001) **b.**

In patients within the HKLC criteria, the OS after the diagnosis of HCC was better among the patients undergoing surgery compared with the patients who received TACE (3-year survival rates, 52.0% vs. 36.5%; 5-year survival rates, 36.3% vs. 14.4%; *P* < 0.001, Figure 1a). Among the patients outside the HKLC criteria, the OS rate in the surgery group was also significantly higher compared with the rate in the TACE group (3-year survival rates, 57.6% vs. 40.1%; 5-year survival rates, 33.3% vs. 10.5%; *P* = 0.001, Figure 1b).

**Figure 2:**
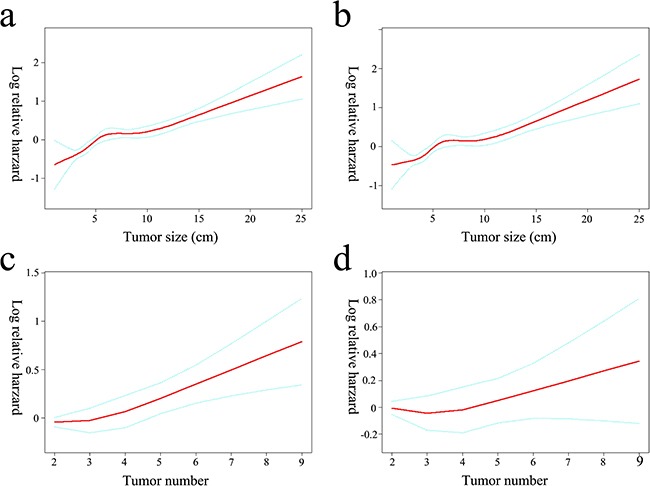
Log-relative risk of death related to tumor size and number The crude **a.** and adjusted **b.** relationship between tumor size and the log-relative risk of death. An inflection of tumor size fixed at 5 cm that gave the maximum likelihood in the 2-piecewise linear regression model (crude P = 0.032, adjusted P = 0.007, respectively). The log-relative risk of death increased linearly with tumor numbers if no covariates were adjusted (OR = 1.1, *P* < 0.001) **c.** No significant association was observed between tumor number and the relative risk of death after adjusting for tumor size and other covariates (OR = 1.0, *P* = 0.615) **d.**

After excluding 404 patients receiving TACE, baseline characteristics of the remaining 941 patients undergoing surgery were described in Table 3. The effect of size demonstrated a non-linear behavior in which the increment was over zero for up to approximately 5 cm (Figure 2a). Even after adjusting for other covariates, the relationship did not change significantly (Figure 2b). Incidentally, such a result supported a size of 5 cm as the accepted cut-off for the criteria currently used for hepatectomy candidacy. Before adjusting the other factors, the survival risk increased with a tumor number over three (Figure 2c). However, after adjustment for other covariates, the relationship between the tumor number and survival risk was no longer significant (Figure 2d).

**Table 3:** Baseline characteristics of 941 patients underwent surgery*

Characteristics	
Age, > 60 years	197 (20.9)
Sex, female	93 (9.9)
Total bilirubin, > 20.4 μmol/L	145 (15.4)
Serum ALT, > 42 u/L	356 (37.8)
Serum GGT, > 54 u/L	647 (68.7)
Serum AFP, > 20 ng/mL	676 (71.8)
Tumor size, cm	5.5 (3.8 - 8.5)
Tumor number	2 (2 - 3)
With tumor thrombus	186 (19.8)
Tumor location, more than one lobe	418 (44.4)
Without tumor capsule	470 (49.9)
Tumor differentiate, III-IV	310 (32.9)

* Continuous variables are presented as median (interquartile range), categorical variables as numbers (%).

Abbreviations: ALT: alanine aminotransferase; GGT, gamma-glutamyl transferase; AFP, alpha-fetoprotein.

Additional exploratory subgroup analyses regarding the effects of tumor thrombus and tumor number on the association between tumor size and OS are shown in Table 4. The predictive capability of tumor size on survival was significantly influenced by tumor thrombus even after adjustment for other covariates (adjusted *P* for interaction = 0.041). In contrast, the predictive capability of tumor size on survival was not influenced by tumor number (adjusted *P* for interaction = 0.258). After adjustments for other covariates in model I and model II, this trend still existed.

**Table 4:** Stratified analysis of factors affecting the correlation between tumor size and overall survival

	Tumor size	No.	Crude OR (95% CI), *P*	Model I^&^	Model II^&^
**Tumor thrombus**					
Without	≤ 5	430	Ref.	Ref.	Ref.
	> 5	325	1.7 (1.4, 2.0)	1.6 (1.4, 1.9)	1.6 (1.3, 1.9)
With	≤ 5	32	1.6 (1.0, 2.6)	1.6 (1.0, 2.4)	1.5 (1.0, 2.4)
	> 5	154	1.7 (1.4, 2.1)	1.5 (1.1, 2.0)	1.4 (1.1, 1.9)
P for interaction			0.048*	0.044*	0.041*
**Tumor number**					
≤ 3	≤ 5	428	Ref.	Ref.	Ref.
	> 5	396	1.6 (1.4, 1.9)	1.6 (1.3, 1.9)	1.5 (1.3, 1.8)
> 3	≤ 5	34	0.9 (0.6, 1.4)	0.8 (0.5, 1.3)	0.9 (0.7, 1.3)
	> 5	83	1.9 (1.5, 2.6)	1.8 (1.4, 2.4)	1.8 (1.3, 2.3)
*P* for interaction			0.223	0.227	0.258

* Significant difference.

Abbreviations: OR: odds ratio; CI: confidence interval

Crude: without adjustment.

Model I: with adjustment of age, sex, alanine aminotransferase; gamma-glutamyl transferase; alpha-fetoprotein and total bilirubin.

Model II: with adjustment of age, sex, alanine aminotransferase; gamma-glutamyl transferase; alpha-fetoprotein, total bilirubin, tumor capsule, location and differentiation.

Due to the presence of interactions, the effect of size could not be represented integrally and therefore was shown for the stratified multivariate Cox proportional-hazards regression model based on whether tumor thrombus is present (Table 5). In patients with tumor thrombus, tumor size > 5 cm was a significant risk factor for survival (OR = 1.7, *P* < 0.001). In addition, a higher degree of tumor differentiation was also marginally significant (OR = 1.2, *P* = 0.071). However, in patients without tumor thrombus, tumor location in more than one lobe was the only independent prognostic factor (OR = 2.1, *P* < 0.001).

**Table 5:** Multivariate Cox regression analyses of the overall survival with clinical characteristics

	With tumor thrombus	*P*	Without tumor thrombus	*P*
	OR(%95 CI)		OR (%95 CI)	
AFP, ng/mL (> 20 vs. ≤ 20)	1.1 (0.9, 1.3)	0.479	1.1 (0.7, 1.7)	0.746
Tumor size, cm (> 5 vs. ≤ 5)	1.7 (1.4, 2.0)	<0.001*	0.9 (0.6, 1.5)	0.717
Tumor number (> 3 vs. ≤ 3)	1.0 (0.8, 1.3)	0.992	1.3 (0.8, 2.1)	0.328
Tumor location (more than one vs. one lobe)	1.0 (0.8, 1.2)	0.673	2.1 (1.5, 3.0)	<0.001*
Tumor capsule (absent vs. present)	1.1 (0.9, 1.3)	0.261	1.0 (0.7, 1.5)	0.928
Tumor differentiate (III-IV vs. I-II)	1.2 (1.0, 1.4)	0.071	1.1 (0.8, 1.6)	0.610

* Significant difference.

Abbreviations: OR: odds ratio; CI: confidence interval

Adjusted: age sex, alanine aminotransferase; gamma-glutamyl transferase and total bilirubin.

Abbreviations: AFP, alpha-fetoprotein

**Figure 3:**
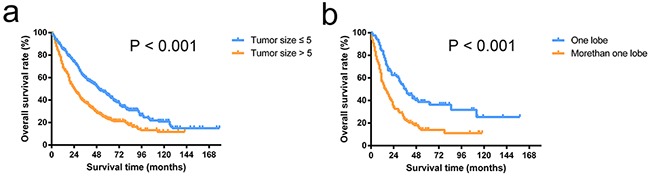
Overall survival curves of patients stratified by tumor thrombus status The overall survival rates of patients with a tumor size of < 5 cm were significantly higher than the rates of patients with a tumor size > 5 cm in patients without tumor thrombus (*P* < 0.001) **a.** The overall survival rates of patients with tumors located in one lobe were significantly higher than in patients with tumors located in more than one lobe in patients with tumor thrombus (*P* < 0.001) **b.**

For patients without tumor thrombus, OS after the operation was better among patients with tumor size ≤ 5 cm compared with those with tumor size > 5 cm (median survival time, 50 vs. 24 months; *P* < 0.001, Figure 3a). For patients with tumor thrombus, OS after operation was better among patients with a tumor location in one lobe compared with those with tumor locations in more than one lobe (median survival time, 37 vs. 15 months; *P* < 0.001, Figure 3b).

## DISCUSSION

In the current study, we found that HBV-related HCC patients with multiple tumors who received surgery could achieve better long-term survival than patients who received TACE treatment, regardless of whether they met the HKLC criteria. This finding suggests that more aggressive standards applied in the treatment of these HBV-related HCC patients are meaningful, although the relevant parameters regarding the HKLC criteria need to be improved. After analyzing the parameters constituting the HKLC criteria, we found that tumor number is not a very good predictor of postoperative survival in patients with multiple tumors. In addition, the state of tumor thrombus demonstrated a significant interaction with tumor size on postoperative survival. In patients without tumor thrombus, tumor size > 5 cm was an independent risk factor of postoperative survival. In patients without tumor thrombus, the patient survival was mainly influenced by the tumor location.

At present, the BCLC system is still one of the most widely accepted grading systems, although many surgery centers do not fully comply with the principles of this system. The majority of HCC occurs in Asia-Pacific areas, and the main cause of HCC in this region is conic HBV infection. The BCLC system's guiding role in HBV-related HCC patients remains controversial [3–5]. However, the surgical risk of patients with multiple tumors is the high risk of tumor recurrence following surgery. With advances in surgical skills and examination techniques, many relapsed patients who have access undergo a second or more repeat hepatectomies. The results from related studies indicate that second or more repeat hepatectomy is a feasible and effective choice for treating the repeat recurrence of HCC and offers satisfactory long-term outcomes [6–9]. A recent multicenter study showed that nearly 70% of patients who underwent hepatic resection are “non-ideal candidates” [10]. In the current study, we found that patients with multiple tumors could achieve a better outcome if they received hepatic resection following the HKLC criteria. This result was previously validated from another study also based on a Chinese population [11]. Some studies from western countries also suggested that multiple tumors is not an absolute contraindication for curative resection [12, 13].

In addition to different etiology and demographic characteristics, cancer genetic heterogeneity is an ignored factor that leads to inconsistent surgical outcomes in patients with multiple tumors. There are differences between patients and between tumor nodules in the same patient, and even within a single tumor nodule [14]. For example, previous studies using whole-genome sequencing examining each nodule in one patient with multiple tumors identified different driver mutations in each nodule [15, 16]. The intratumoral heterogeneity might reflect the existence of distinct pools of cancer stem-like cells that exhibit different tumorigenicities and independent genomic evolution [17]. Therefore, if multifocal HCC develops as a consequence of intrahepatic metastases of the same primary cancer, such patients should belong to an advanced stage, which would lead to poor surgical outcomes. However, if multiple tumors arise either synconously or metaconously as primary tumors (multicentric occurrence), such patients might still be classified as early stage, and surgical removal could achieve satisfactory results. These uncertainties made prognostication after surgery very difficult for individual patients and reasonably explained the results that no significant association was found between tumor number and postoperative survival in patients with multiple tumors.

The mechanisms of tumor thrombus formation have not been well clarified, although the presentation of macroscopic tumor thrombus indicates that HCC developed in an advanced stage is widely acknowledged [18, 19]. At present, there is no concrete evidence for establishing an optimal treatment strategy for patients with tumor thrombus. In the BCLC staging system, the only proposed treatment option for patients with tumor thrombus is sorafenib [20]. However, the HKLC staging system recommends that patients with intrahepatic venous invasion undergo surgery as long the tumor size ≤ 5 cm and tumor nodules ≤ 3. Although these groups of patients have been reported to have a poor prognosis [21], studies from both the West and the East have clearly shown that hepatic resection can be performed safely and effectively in HCC patients with major vascular invasion [22, 23]. In the current study, we verified the effectiveness of aggressive surgical treatment and found that the postoperative survival was affected by the location of the multiple tumors rather than the tumor size in patients with tumor thrombus. One reasonable and easily accepted explanation is that, for patients with tumor thrombus, if the tumor located in a different lobe, these tumors are more likely to spread tough the blood. After the tumors have spread from the original position, a poor prognosis of surgery is widely accepted [21].

After several decades of development in anesthesia and surgical techniques, many surgeons have tried to expand the criteria for hepatectomy for HCC patients. The HKLC, derived from Asian populations, therefore has a natural advantage to guide treatments of HBV-related HCC patients. This study focused on the HKLC standard of surgery indications for patients with multiple tumors. We found that the tumor number had no influence on postoperative survival after adjusting for tumor size in patients with multiple tumors. Therefore, the enrolled tumor size and number as independent parameters in the HKLC criteria still must be determined in the future. In addition, data on tumor characteristics were collected from postoperative histopathology reports. It is known that preoperative imaging reports fail to predict tumor size, number and thrombus in approximately 25–35% of patients due to understaging and overstaging [24, 25]. We also enrolled other parameters such as blood test indicators, tumor number and differentiation grades in the analysis, which could potentially provide a reason for the inconsistency between the current study and the HKLC system.

There were several limitations of this study. First, due to the nature of the retrospective study, there would be potential bias that might prevent definite conclusions from being drawn. Although we used propensity score methods and multivariable statistical methods to estimate these conclusions while adjusting for confounding, results from current study still need more randomized controlled trials to validate and more studies on the mechanism of tumorigenesis to support, before changing the HKLC criteria. Second, we found that relying solely on the number of tumors to distinguish multiple tumors as metastasis or multi-center occurrence is not accurate. However, an alternative simple and effective approach needs to be further studied.

The present study validated the effectiveness of more aggressive surgical treatment for patients with multiple tumors and even with intrahepatic venous invasion. Such patients are classified as intermediate stage (B) or advanced stage (C) according to BCLC staging and would be considered only for noncurative options, such as TACE or systemic treatment. However, limited to accuracy and feasibility of preoperative examination, the criteria raised in the HKLC system remain to be further adjusted and verified. With the elucidation of mechanisms of multiple tumors in the future, more HCC patients could achieve long-term survival outcomes tough surgical treatment.

## MATERIALS AND METHODS

Between January 2004 and December 2012, a total of 5382 adult patients with HCC underwent curative resection in Zhongshan Hospital. In the current study, only patients meeting the following criteria were enrolled in the study population: (1) Eastern Cooperative Oncology Group performance status 0-1; (2) Child–Pugh grade A; (3) no exhepatic metastasis; (4) HCC as confirmed by postoperative histology; (5) tumor number ≥ 2 and confirmed intraoperatively and postoperatively; and (6) patients underwent radical resection. The radical resection criterion was the same as previously described [26]. Generally speaking, patients with a single tumor or tumors with diffuse distribution as well as those who received only palliative tumor resection or other intraoperative or postoperative adjuvant anti-tumor treatments were excluded from this study. Following these inclusion and exclusion criteria, a total of 941 patients receiving surgery were included in the current study. Then, we followed the same criteria described above for radiologic appearance combined with the AFP level instead of a histology diagnosis and enrolled 404 patients who received TACE during the same period in the current study. Owing to the shortage of donors and high cost, liver transplantation is still rare in China [27]. In the current study, we did not include this kind of treatment protocol. The demographic and clinical characteristics of the 1345 cases before matching are summarized in Supplementary Table S1.

This retrospective study evaluated patient data from a database collection retrieved from our electronic medical records and anonymized prior to the analysis. The study protocol followed the ethical guidelines of the 1975 Declaration of Helsinki (as revised in Brazil in 2013) and was approved by the Zhongshan Hospital Research Ethics Committee.

### Surgery

In our center, patients were staged preoperatively by abdominal ultrasonography, contrast computed tomography or magnetic resonance imaging scanning. The surgical indications for HCC were determined by the surgical team according to a decision tree based on the serum bilirubin level, the remaining liver volume, the presence or absence of ascites, and the patient's performance status, as described previously [12]. For patients who underwent operation, the tumor number was further evaluated by visual inspection, manual palpation, and intraoperative ultrasonography, as previous described [28, 29]. Newly detected HCC was resected whenever possible.

### Clinicopathological factors

In this study, all patients tested positive for hepatitis B surface antigen or had detectable levels of HBV DNA in their serum by polymerase chain reaction (PCR)–based methods. If blood test results showed the hepatitis B virus replication, patients received antiviral treatment before surgical operation. Generally, nucleotide analogues therapy carried out immediately. Even after the surgery, patients continued taking nucleotide analogues and received evaluation of HBV replication to ensure serum HBV levels were maintained at 5.0 log copies/ml or less. Other clinical factors that are potentially related to HCC patients' prognosis were selected on the basis of our previous studies [30, 31]. Tumor location was categorized by multiple tumors' location (only one lobe or more than one lobe) based on Couinaud's nomenclature [32]. Continuous variables as total bilirubin, serum alanine aminotransferase, GGT, and AFP were categorized by using the cut-off values provided by clinical references, as tumor size, tumor number were categorized by presenting similar cut-off values used in HKLC criteria [2], as age was categorized by an accepted cut-off point used for clinical decision-making as previous study [33].

### Follow up

All patients were periodically observed at follow-up to detect the recurrence of HCC at the clinic. Generally, testing for blood tumor markers and ultrasonography were performed every 2 months for the first year and at least every 3 months thereafter. In addition, an enhanced magnetic resonance imaging scan of the abdomen was performed every 6-12 months.

Whenever recurrence was confirmed, further treatment was immediately administered. If the recurrent tumor was localized, a second liver resection, percutaneous ethanol injection, or radio frequency ablation was suggested based on the specific circumstances. If the recurrent tumor was multiple or diffused, TACE was the preferred choice; if the tumor had metastasized to the lymph nodes or bone, external radiotherapy was administered. The overall survival (OS) time was defined as the time from the date on which the first treatment started to the date of death or last contact for surviving patients. Follow-up data for all patients were summarized at the end of December 2014, with a median observation time of 29 months.

### Statistical analysis

Because this study was nonrandomized and observational, propensity score methods were used to reduce the bias in estimating treatment effects and allow investigators to reduce the likelihood of confounding [34]. We computed the propensity score by using multiple logistic regression with the dependent variable receiving surgery or TACE. The independent variables were tumor size, number, thrombus, location, total bilirubin, AFP and ES grading. Patients were matched by a 1:1 ratio using the nearest neighbor method with a caliber of 0.2. In patients within HKLC criteria, after excluding 27 patients without appropriate pairs, 237 patients received TACE were matched to 237 patients received surgery. In patients outside HKLC criteria, after excluding 23 patients without appropriate pairs, 117 patients received TACE were matched to 117 patients received surgery.

We first compared the baseline characteristics of the two propensity-matched groups stratified by HKLC criteria using Student t-tests for continuous variables and Chi-square tests for categorical data (Table 1). A multivariate Cox proportional-hazards regression model was used to examine the independent risk factors associated with patient survival (Table 2). The OS was calculated using the Kaplan–Meier method and compared using the log-rank test (Figure 1). Then, we described baseline characteristics of 941 patients underwent surgery in Table 3. The relationships between tumor size or tumor number and patient survival were explored by a smoothing plot, with or without an adjustment for potential confounders (Figure 2). An exploratory stratified analysis was performed, and the P value for the interaction was calculated from the log likelihood ratio test comparing two nested models (Table 4). Finally, a multivariate Cox proportional-hazards regression model was used again to examine the independent risk factors associated with OS in each subgroup (Table 5). The OS in different groups was also calculated using the Kaplan–Meier method and compared by the log-rank test (Figure 3).

All of the data were double-entered and then exported to tab-delimited text files. The missing data on prognostic factors were filled in by multiple imputation using a stochastic switching regression approach with 5 repeated imputations [35]. P values < 0.05 were considered statistically significant. The statistical analysis was performed using R (http://www.R-project.org, version 3.2.2).

## SUPPLEMENTARY TABLE


