# Supplementary material for: Is the Hong Kong Liver Cancer staging system the best guide for hepatitis B virus-related hepatocellular carcinoma patients with multiple tumors?
Source: Oncotarget. 2016 Jun 13;7(32):51598–607. doi: 10.18632/oncotarget.9956 (PMC5239499; doi:10.18632/oncotarget.9956)
Supplement: Supplementary file 1 [file oncotarget-07-51598-s001.pdf]

Is the hong kong liver cancer staging system the best guide for hepatitis B virus-related hepatocellular carcinoma patients with multiple tumors?

SUPPLEMENTARY TABLE

Supplementary Table S1: Base line characteristics before matching related to Surgery and TACE stratified by HKLC criteria

	Within HKLC criteria		*P*	Outside HKLC criteria		*P*
	Surgery (n = 720)	TACE (n = 264)		Surgery (n = 221)	TACE (n = 140)	
Age, years			0.119			<0.001*
≤ 60	558 (77.5)	192 (72.7)		186 (84.2)	96 (68.6)	
> 60	162 (22.5)	72 (27.3)		35 (15.8)	44 (31.4)	
Sex			0.496			0.518
Female	66 (9.2)	28 (10.6)		27 (12.2)	14 (10.0)	
Male	654 (90.8)	236 (89.4)		194 (87.8)	126 (90.0)	
Total bilirubin, µmol/L			0.989			0.011*
≤ 20.4	603 (83.8)	221 (83.7)		193 (87.3)	108 (77.1)	
> 20.4	117 (16.2)	43 (16.3)		28 (12.7)	32 (22.9)	
Serum ALT, u/L			0.859			0.126
≤ 42	451 (62.6)	167 (63.3)		134 (60.6)	96 (68.6)	
> 42	269 (37.4)	97 (36.7)		87 (39.4)	44 (31.4)	
Serum GGT, u/L			0.226			0.689
≤ 54	259 (36.0)	84 (31.8)		35 (15.8)	20 (14.3)	
> 54	461 (64.0)	180 (68.2)		186 (84.2)	120 (85.7)	
Serum AFP, ng/mL			0.185			0.921
≤ 20	228 (31.7)	72 (27.3)		37 (16.7)	24 (17.1)	
> 20	492 (68.3)	192 (72.7)		184 (83.3)	116 (82.9)	
Tumor size, cm			0.278			<0.001*
≤ 5	453 (62.9)	176 (66.7)		9 (4.1)	32 (22.9)	
> 5	267 (37.1)	88 (33.3)		212 (95.9)	108 (77.1)	
Tumor number			<0.001*			<0.001*
≤ 3	695 (96.5)	212 (80.3)		129 (58.4)	48 (34.3)	
> 3	25 (3.5)	52 (19.7)		92 (41.6)	92 (65.7)	

(Continued)

	Within HKLC criteria			Outside HKLC criteria		
	Surgery	TACE	*P*	Surgery	TACE	*P*
	(n = 720)	(n = 264)		(n = 221)	(n = 140)	
Tumor thrombus			0.017*			0.554
Without	692 (96.1)	244 (92.4)		63 (28.5)	44 (31.4)	
With	28 (3.9)	20 (7.6)		158 (71.5)	96 (68.6)	
Tumor location			0.625			0.436
One lobe	427 (59.3)	152 (57.6)		96 (43.4)	55 (39.3)	
More than one lobe	293 (40.7)	112 (42.4)		125 (56.6)	85 (60.7)	

* Significant difference.
Abbreviations: TACE, transcatheter arterial chemoembolization; ALT: alanine aminotransferase; GGT, gamma-glutamyl transferase; AFP, alpha-fetoprotein.
